# A Systematic Review Showing the Comparative Efficacy and Safety of Endoscopic Submucosal Dissection Versus Endoscopic Mucosal Resection in the Management of Colorectal Neoplasia

**DOI:** 10.7759/cureus.109031

**Published:** 2026-05-17

**Authors:** Achsah Raj Chandralekha, Maryam Kayani, Aleena Javed, Akanksha Sharma, Anum Naimat, Shravani Yedulapuram, Seena Sugathan, Alaa Osman, Bashir Imam, Darwin A Espiritu, Abirami Rajendiran, Joshua Elefteratos, Ramsha Ali

**Affiliations:** 1 Department of Medicine, Christian Medical College and Hospital, Ludhiana, IND; 2 Department of Internal Medicine, Shifa International Hospital, Islamabad, PAK; 3 Department of Medicine, Combined Military Hospital (CMH) Lahore Medical College and Institute of Dentistry, Lahore, PAK; 4 Faculty of Medicine, Christian Medical College and Hospital, Ludhiana, IND; 5 Department of Internal Medicine, FMH College of Medicine & Dentistry, Lahore, PAK; 6 Faculty of Medicine, Sri Ramachandra Institute of Higher Education and Research, Chennai, IND; 7 Department of Acute Medicine, North Manchester General Hospital, Manchester, GBR; 8 Department of Medicine, Lebanese University, Beirut, LBN; 9 Department of Pediatrics, University of Pittsburgh Medical Center, Coudersport, USA; 10 Department of General Internal Medicine, University Hospitals Coventry and Warwickshire NHS Trust, Coventry, GBR; 11 Department of Internal Medicine, Texas Tech University Health Sciences Center, Paul L. Foster School of Medicine, El Paso, USA; 12 Faculty of Medicine, Montefiore Medical Center, Wakefield Campus, New York, USA; 13 Department of Medicine and Surgery, Peoples University of Medical and Health Sciences, Nawabshah, PAK

**Keywords:** colorectal neoplasms, endoscopic mucosal resection, endoscopic submucosal dissection, postoperative complications, treatment outcomes

## Abstract

Colorectal neoplasia contributes substantially to the global burden of disease, and selecting the most appropriate endoscopic treatment is essential to reduce recurrence and avoid surgery. This systematic review focused exclusively on direct comparisons of endoscopic mucosal resection (EMR) and endoscopic submucosal dissection (ESD) for colorectal neoplasia while excluding non-colorectal lesions, single-arm studies, rectal neuroendocrine tumors, and other non-comparable interventions (studies without direct EMR versus ESD comparison). A systematic search of PubMed, Embase, and Scopus from inception to June 2025 was performed in accordance with the PRISMA guidelines. Six reviewers independently screened studies, and 17 comparative studies involving 3790 lesions were included. Data were extracted for tumor size, procedure time, en bloc resection, complete RO resection, recurrence, and major complications, including perforation and delayed bleeding. Risk of bias was assessed using the Newcastle-Ottawa Scale for observational studies and the Cochrane Risk of Bias 2 (RoB 2) tool for randomized trials. Across included studies, ESD was used for larger lesions, required longer procedure times, and achieved higher en bloc and R0 resection rates than EMR. ESD also demonstrated lower recurrence, whereas perforation and delayed bleeding were generally more frequent after ESD. EMR remained faster and was associated with a lower perforation risk, particularly in smaller and less complex lesions. Overall, ESD provides superior oncologic resection quality and lower recurrence but at the cost of greater technical burden and a higher complication profile.

## Introduction and background

Colorectal neoplasia refers to abnormal tissue growth in the colon and rectum. It is a major global health concern because of its potential to progress to colorectal cancer, which is the third most commonly diagnosed cancer and the second leading cause of cancer-related deaths worldwide [1]. Early detection and appropriate endoscopic treatment play an important role in reducing recurrence, limiting progression, and avoiding unnecessary surgery.

Advances in therapeutic endoscopy have made endoscopic mucosal resection (EMR) and endoscopic submucosal dissection (ESD) the two most frequently used minimally invasive approaches for colorectal neoplasia [2]. EMR is widely used because it is simpler, faster, and generally safer, especially for smaller lesions less than 20 mm. ESD, by contrast, permits en bloc resection regardless of lesion size, facilitates more accurate histopathologic assessment, and may reduce recurrence, but it is technically time-intensive and associated with a higher complication risk [2,3].

Understanding the comparative efficacy and safety of EMR and ESD is essential for optimizing clinical decision-making and patient outcomes. The choice between these techniques influences recurrence, complication rates, resource utilization, and overall quality of care [3,4]. Although several reviews have compared these modalities, there remains no universally accepted standard approach for all colorectal neoplastic lesions, particularly when lesion size, morphology, malignant potential, and local expertise differ [3,4]. This systematic review was undertaken to provide an updated clinically focused comparison of EMR and ESD in the management of colorectal neoplasia.

## Review

Materials and methods

Study Design and Objectives

This study was conducted as a systematic review in accordance with the PRISMA guidelines [5] and was prospectively registered in the International Prospective Register of Systematic Reviews (PROSPERO) (CRD420251124096). The population of interest comprised adults aged 18 years or older with colorectal neoplasia, including adenomas and early-stage colorectal cancers, who were eligible for endoscopic resection. The intervention was ESD, and the comparator was EMR. The primary outcomes were en bloc resection rate and complete RO resection rate. Secondary outcomes included local recurrence, procedure duration, and complication rates, particularly perforation and delayed bleeding.

Search Strategy

A comprehensive literature search was performed in PubMed, Scopus, and Embase from database inception through June 2025. The search strategy used MeSH and free text terms related to colorectal neoplasia, endoscopic submucosal dissection, endoscopic mucosal resection, ESD, and EMR, combined with Boolean operators such as AND and OR. Only studies published in the English language were considered.

Inclusion Criteria

Eligible studies included adults with colorectal neoplasia and directly compared ESD with EMR. Studies had to report at least one clinically relevant outcome such as en bloc resection, R0 resection, recurrence, perforation, bleeding, procedure time, or overall complications. Randomized controlled trials, cohort studies, case control studies, and case series with at least five patients were eligible.

Exclusion Criteria

Studies were excluded if they focused on non-colorectal lesions such as gastric, esophageal, or duodenal neoplasms, if they evaluated only one intervention without direct comparison, or if they included more than two intervention groups. Editorials, letters, conference abstracts, commentaries, review articles without primary clinical data, case reports, case series with fewer than five patients, animal studies, in vitro studies, and non-English publications were also excluded.

Study Selection Process

All potentially relevant citations were imported into Rayyan (Cambridge, MA) for structured and blinded screening. Titles and abstracts were reviewed independently by six reviewers, and full-text screening was performed for studies meeting the inclusion criteria. Disagreements during screening and selection were resolved by reviewer consensus.

Results

Study Selection

**Figure 1:**
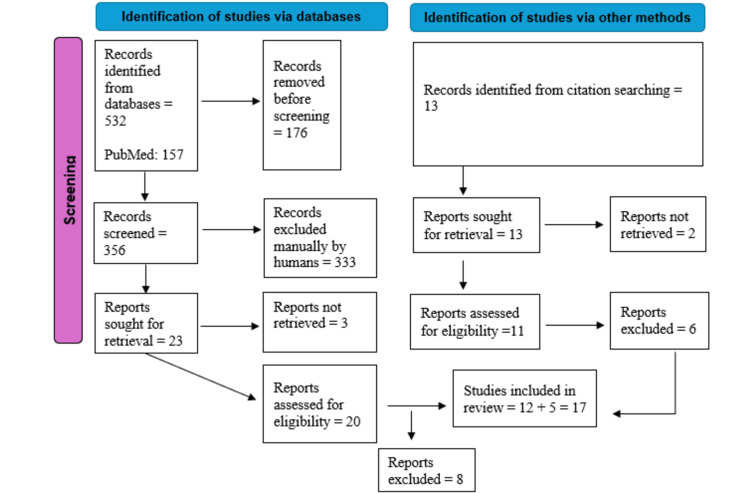
PRISMA flow diagram showing study identification, screening, eligibility assessment, and final inclusion of the studies.

A total of 532 records were identified through database searches, including PubMed (n = 157), Scopus (n = 300), and Embase (n = 75). An additional 13 records were identified through citation searching. After removal of 176 duplicate records, 356 studies remained for title and abstract screening, and 333 were excluded by human reviewers. Twenty-three reports were sought for full text retrieval from the database search, of which three could not be retrieved. Twenty database-derived reports were assessed for eligibility, and eight were excluded. All 13 citation-derived records were sought for retrieval; two could not be retrieved, 11 were assessed for eligibility, and six were excluded. Ultimately, 17 studies were included in the final review. The study selection process is summarized in Figure 1.

Study Characteristics

Data extraction was performed independently by multiple reviewers. Extracted variables included lesion count, histologic diagnosis, tumor size, procedure time, en bloc resection, complete RO resection, perforation, delayed bleeding, and recurrence. When both unmatched and propensity-matched analyses were available, data from the propensity-matched cohort were preferentially extracted because these analyses were considered less confounded [6-8].

Outcome Definition

En bloc resection was defined as the removal of the lesion in a single intact specimen rather than in multiple fragments. R0 resection indicated complete tumor removal with microscopically negative margins. Procedure time was measured from initial lesion marking to completion of resection. Perforation was defined by direct endoscopic visualization of a full-thickness mural defect or by radiologic evidence of free air. Local recurrence was defined as reappearance of a lesion with the same histologic type at the site of the original resection during follow-up [9,10]. Delayed bleeding referred to melena, hematochezia, or clinically significant post-procedural bleeding occurring within 0 to 14 days after resection [11].

Risk of Bias Assessment

Risk of bias was assessed for all 17 included studies. The review included 15 cohort studies, one case-control study, and one randomized controlled trial (Table 1).

**Table 1:** Characteristics of included studies. Abbreviations: EMR, endoscopic mucosal resection; ESD, endoscopic submucosal dissection; EMR-P, precutting EMR; UEMR, underwater EMR; C-EMR, circumferential-incision EMR; LST, laterally spreading tumor; NR, not reported in the indexed English abstract or bibliographic record.

Study	Country	Design	Setting/period	Population	Comparator(s)	Sample size	Key notes
Saito et al. [12]	Japan	Retrospective comparative cohort	Single-center; 1995-2009	Large colorectal tumors (generally ≥20 mm)	EMR vs. ESD	EMR 228; ESD 145	Landmark early direct comparison; larger lesions increasingly treated with ESD.
Toyonaga et al. [13]	Japan	Retrospective comparative study	Single-center; period NR	Early-stage colorectal tumors	EMR with small incision/simplified ESD/ESD	EMR-family 24; ESD-family 512	Three-arm variant study; included because the manuscript synthesizes EMR-family vs. ESD-family comparative data.
Tajika et al. [14]	Japan	Retrospective cohort	Single-center; period NR	Large colorectal tumors	EMR vs. ESD	EMR 104; ESD 85	Compared outcomes in large lesions; higher en bloc/R0 rates favored ESD.
Kobayashi et al. [15]	Japan	Matched case-control study	Single-center; 2005-2009	Colorectal tumors	EMR vs. ESD	EMR 56; ESD 28	Matching reduced confounding between approaches.
Terasaki et al. [9]	Japan	Retrospective comparative cohort	Single-center; 2006-2009	Laterally spreading tumors >20 mm	EMR/piecemeal EMR vs. ESD/hybrid ESD	EMR-family 178; ESD-family 89	Variant-based comparative analysis in large LSTs.
Lee et al. [10]	South Korea	Retrospective comparative cohort	Single-center; 2004-2009	Large nonpedunculated colorectal tumors	EMR vs. EMR-precutting vs. ESD	EMR 140; EMR-P 69; ESD 314	Three-arm comparison; manuscript-level synthesis groups EMR-type techniques against ESD.
Kim et al. [16]	South Korea	Retrospective comparative cohort	Single-center; period NR	Colorectal neoplasia	EMR-P vs. ESD with snaring vs. ESD	EMR-P 91; ESD-S 57; ESD 58	Technique-variant study; retained because it directly compares EMR-related and ESD-related methods.
Urban et al. [17]	Czech Republic	Prospective comparative cohort	Single-center; EMR 2010-2011, ESD thereafter	Flat neoplastic rectal lesions	EMR vs. ESD	EMR 30; ESD 27	Western-center rectal series.
Jung et al. [18]	South Korea	Retrospective multicenter cohort	Multicenter; period NR	Colorectal laterally spreading tumors with advanced histology	Endoscopic resection modalities, including EMR and ESD	Total 246 lesions; arm-specific counts NR	Advanced-histology subgroup comparison.
Zou et al. [19]	China	Comparative cohort	Single-country study; period NR	Colorectal laterally spreading tumors	Endoscopic resection modalities, including EMR and ESD	NR from indexed English sources	Included study identified from comparative LST literature; full arm-level details were not available in indexed English records.
Cui et al. [20]	China	Comparative cohort	Single-country study; period NR	Colorectal laterally spreading tumors	Endoscopic treatment modalities, including EMR and ESD	NR from indexed English sources	Included study identified from comparative LST literature; indexed metadata were limited.
Li et al. [21]	China	Retrospective multicenter study	Six hospitals; 2007-2017	Colorectal laterally spreading tumors	EMR vs. ESD	Total 653 patients; arm-specific counts NR in abstract	Large multicenter long-term outcomes study.
Ohmori et al. [6]	Japan	Retrospective propensity-matched pilot study	Single-center; 2013-2019	Residual/recurrent colorectal neoplasms after prior endoscopic resection	Underwater EMR vs. ESD	Total 51 lesions; matched-arm counts NR in abstract	Specialized recurrent-lesion comparison.
Oh et al. [7]	South Korea	Retrospective propensity-matched cohort	Single-center; 2014-2019	Flat colorectal lesions 20-30 mm	Precutting EMR vs. ESD	After matching: 90 vs. 90	Direct comparison of medium-size flat lesions.
Takada et al. [8]	Japan	Retrospective propensity-matched cohort	Single-center; 2014-2019	Nonpedunculated colorectal neoplasms 20-30 mm	Tip-in EMR vs. ESD	After matching: 140 vs. 140	Modern EMR-variant comparison study.
Abdulzhalieva et al. [22]	Russia	Prospective randomized trial	Single-center; period NR	Benign epithelial colorectal neoplasms 20-30 mm	Circumferential-incision EMR vs. ESD	C-EMR 26; ESD 24	Only randomized trial in the included set.
Kouladouros et al. [23]	Germany	Retrospective two-center cohort	Two centers; 2010-2022	Rectal lesions involving the dentate line	EMR vs. ESD	EMR 62; ESD 68	Recent Western comparative series in a technically challenging location.

Observational studies were assessed using the Newcastle-Ottawa Scale (NOS), and most were graded at 7/8 stars, indicating generally good methodological quality [24]. The randomized trial was evaluated with the Cochrane Risk of Bias 2 (RoB 2) tool and was judged to have an overall low risk of bias [25]. A summary of the risk of bias assessment is presented in Table 2.

**Table 2:** Risk of bias assessment summary.

Study design	No. of studies	Assessment tool	Overall assessment reported in this review
Cohort studies	15	Newcastle-Ottawa Scale	Most studies were graded 7/8 stars, indicating generally good methodological quality.
Case-control study	1	Newcastle-Ottawa Scale	Methodological quality was considered acceptable.
Randomized controlled trial	1	Cochrane Risk of Bias 2 (RoB 2)	Overall, low risk of bias.

Sample Size

The 17 included studies reported 3790 lesions in total, including 1858 treated with EMR and 1932 treated with ESD. When reported, study periods ranged from 2003 to 2022, and the included articles were published between 2007 and 2024. EMR sample sizes ranged from 11 to 350 lesions, and ESD sample sizes ranged from 11 to 429 lesions. Fifteen studies were retrospective cohort studies, one was a case control study, and one was a randomized controlled trial.

Histological Diagnosis

Histologic classification was reported in 14 of the 17 studies covering 1520 EMR lesions and 1587 ESD lesions. Among EMR lesions, 1095 (72.03%) were benign and 425 (27.96%) were malignant. Among ESD lesions, 922 (58.09%) were benign and 665 (41.09%) were malignant. These data suggest that EMR was used more often for benign lesions, whereas ESD was more frequently applied to malignant or higher-risk lesions.

Tumor Dimension

Tumor dimensions were reported in 16 of the 17 studies. To standardize reporting, tumor size data were converted to mean ± standard deviation where necessary using the methods described by Wan et al. [26]. The average lesion size was 27.46 mm for EMR and 33.493 mm for ESD. Across studies, mean tumor size ranged from 11.25 ± 5.96 mm to 34.3 ± 15.8 mm in EMR cohorts and from 11.25 ± 6.59 mm to 50.0 ± 24.7 mm in ESD cohorts. This pattern consistently showed preferential use of ESD for larger and more complex lesions.

Procedure Time

**Figure 2:**
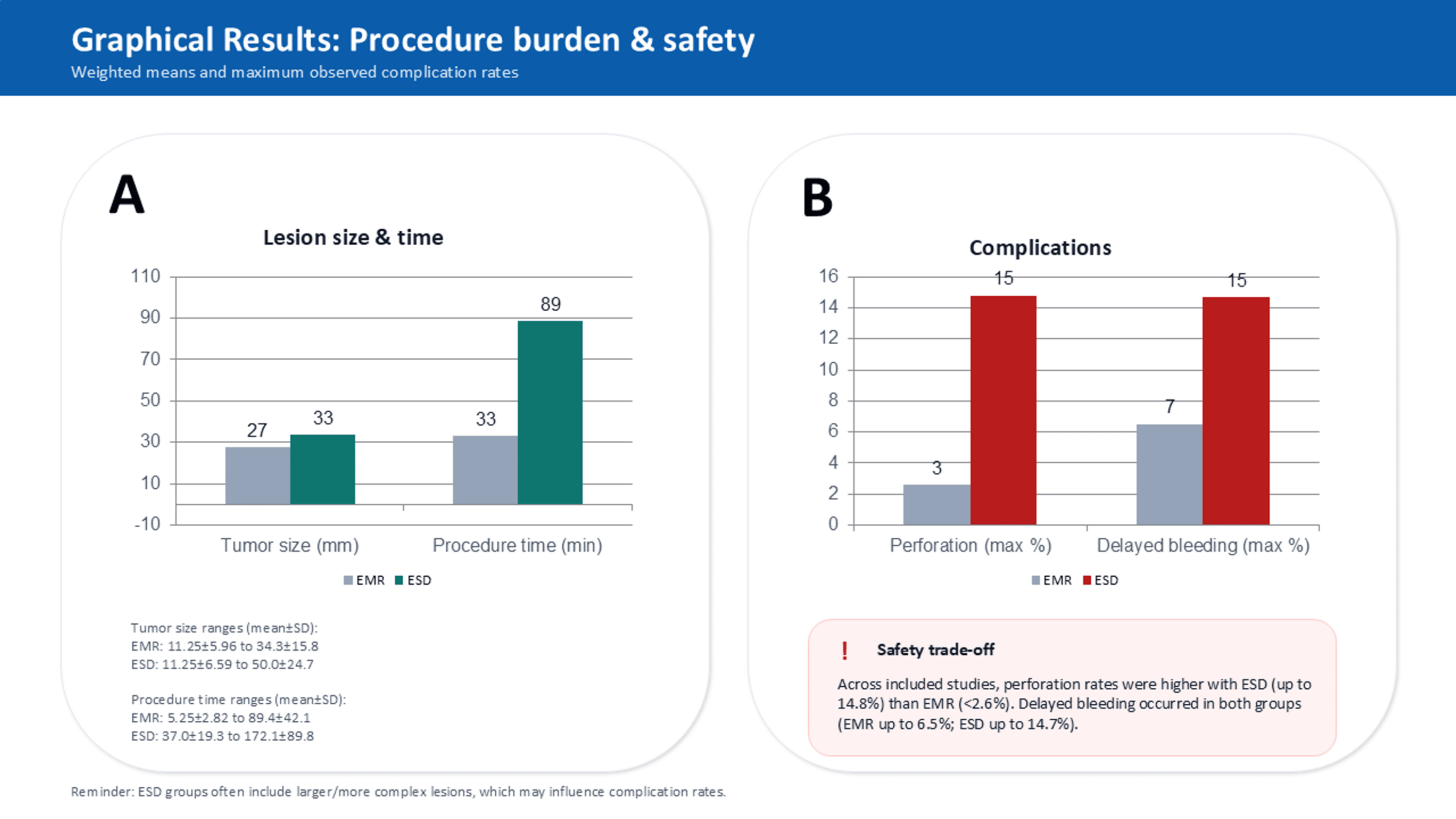
Graphical results summarizing (A) comparative lesion size and procedure time and (B) comparative perforation and delayed bleeding profiles for EMR vs. ESD. Abbreviations: EMR, endoscopic mucosal resection; ESD, endoscopic submucosal dissection. Image credits: Ramsha Ali. Figure prepared using Microsoft PowerPoint (Microsoft Corporation, Redmond, WA).

Procedure time was reported in 15 studies, and direct EMR versus ESD comparisons were available in 13 of them. After standardization to mean ± standard deviation, the weighted average procedure time was 32.914 minutes for EMR and 88.65 minutes for ESD. Reported ranges were 5.25 ± 2.82 to 89.4 ± 42.1 minutes for EMR and 37.0 ± 19.3 to 172.1 ± 89.8 minutes for ESD. Across all included comparative studies, EMR consistently required less time than ESD, reflecting the greater technical complexity of submucosal dissection. Figure 2A summarizes the comparative lesion size and procedure time patterns.

En Bloc Resection

Fifteen studies reported comparative en bloc resection rates. En bloc resection ranged from 61% to 100% in ESD cohorts and from 0% to 100% in EMR cohorts. Abdulzhalieva et al. reported 100% en bloc resection in both groups [22]. Overall, ESD achieved higher en bloc resection rates than EMR.

R0 Resection

Twelve studies compared R0 resection rates between ESD and EMR. Across these studies, R0 resection ranged from 0% to 95.8% for ESD and from 0% to 96.3% for EMR. Two studies found similar rates between techniques.

Perforation

Perforation rates were generally higher in the ESD group, reaching up to 14.8%, compared with less than 2.6% in the EMR group. This finding reflects the deeper dissection plane required for ESD and the technical challenge associated with more advanced lesions.

Delayed Bleeding

Delayed bleeding occurred in both groups but tended to be higher in ESD cohorts, ranging up to 14.7%, compared with up to 6.5% in EMR cohorts. Abdulzhalieva et al. reported no delayed bleeding in patients treated with circumferential incision EMR, suggesting that selected EMR variants may provide a favorable safety profile in appropriate clinical settings [22]. Figure 2B summarizes the comparative perforation and delayed bleeding profiles.

Recurrence Rate

Recurrence rates were consistently lower with ESD, ranging from 0% to 9%, compared with 0% to 41% after EMR. These findings support the superior long-term efficacy of ESD for sustained lesion clearance.

Discussion

Several prior systematic reviews and meta-analyses have compared EMR and ESD for colorectal lesions, but many have synthesized highly heterogeneous lesion types [4,27-33]. Two earlier reviews specifically focused on non-pedunculated colorectal lesions larger than 20 mm that were considered noninvasive and technically resectable [27,28]. Other reviews included more heterogeneous lesion categories, including lesions with different endpoints or more specialized populations [4,29]. By excluding polyps, rectal carcinoids, and non-comparable interventions, the present review aimed to provide a more clinically coherent synthesis focused on colorectal neoplasia of direct oncologic relevance.

Despite these methodological differences, the present findings are consistent with previous evidence. Across the available literature, ESD has repeatedly shown higher en bloc and R0 resection rates and lower recurrence than EMR while also requiring more time and carrying a higher perforation risk [4,27-33]. Despite these margin clearance advantages, prior reviews have not consistently shown a reduction in subsequent surgery, suggesting that lesion biology, staging, and patient selection continue to influence downstream management.

This review also helps contextualize outcomes in Western settings, which remain underrepresented in earlier analyses. Four included studies were from Western centers [17,22,23]. These studies generally reported longer ESD procedure times than many Asian series, and some reported higher perforation rates likely reflecting differences in case selection, procedural volume, and operator experience [17,22,23]. At the same time, strong outcomes from selected Western centers suggest that structured training and experience may be at least as important as geography alone [17,22,23].

Several evidence gaps remain. Most included studies were retrospective, and only one randomized controlled trial was available. Heterogeneity in reporting definitions, lesion selection, and EMR variants limits direct comparison across studies. Future multicenter prospective studies and randomized trials should adopt standardized outcome definitions and explore how structured training pathways, lesion complexity, and newer EMR variants affect comparative effectiveness.

An important limitation of this review is the potential for selection bias and confounding by indication because ESD was more frequently used for larger, more complex, and histologically advanced lesions, whereas EMR was generally applied to smaller and lower-risk lesions. Although propensity-matched analyses were preferentially extracted when available, heterogeneity in study design and reporting limited formal subgroup comparison between matched and unmatched cohorts. Consequently, some observed differences in efficacy and complication rates may partially reflect baseline lesion characteristics rather than procedural effects alone.

## Conclusions

EMR and ESD each offer important advantages in the management of colorectal neoplasia. In this review, ESD provided higher en bloc and complete R0 resection rates with lower recurrence, but it was also associated with longer procedure times and a higher risk of perforation with delayed bleeding. EMR remained faster and generally safer with respect to perforation, although it was less effective for the complete resection of larger or more advanced tissue.

These findings support tailoring the choice of endoscopic technique to lesion size, morphologic complexity, histologic suspicion, and the availability of operator expertise. In centers with appropriate experience and resources, ESD should be strongly considered for lesions in which en bloc and margin-negative resection are especially important. Additional prospective multicenter research is needed to validate these findings and support the development of standardized evidence-based pathways for colorectal endoscopic therapy.
